# Adaptative and ancient co-evolution of integrons with Xanthomonas genomes

**DOI:** 10.1099/mgen.0.001503

**Published:** 2025-09-17

**Authors:** Elena Colombi, Timothy M. Ghaly, Vaheesan Rajabal, Liam D.H. Elbourne, Michael Gillings, Sasha Tetu

**Affiliations:** 1 School of Natural Sciences, Macquarie University, Sydney, NSW, 2109, Australia; 2 ARC Centre of Excellence in Synthetic Biology, Sydney, NSW, 2109, Australia

**Keywords:** genome evolution, horizontal gene transfer, mobile genetic elements, niche adaptation, plant pathogen

## Abstract

Integrons are genetic elements that facilitate gene acquisition. They have been extensively studied in clinical bacteria, but their evolutionary role in phytopathogens remains underexplored. Here, we analysed complete genomes of *Xanthomonas* species to investigate the origin, distribution and functional dynamics of integrons in this genus. We found that 93% of genomes harboured integrons. The integron-integrase gene *intI* was predominantly located downstream of *ilvD*, indicating an ancestral acquisition of integrons, predating diversification within the genus. Phylogenetic analyses support vertical inheritance of *intI*, with the exception of rare horizontal gene transfer events, notably in *Xanthomonas arboricola*. Despite their widespread presence, full-length *intI* genes and active integron platforms are only retained in some species, especially *Xanthomonas campestris*, which shows high integron gene cassette variability and functional integron activity. In contrast, species such as *Xanthomonas cissicola* and *Xanthomonas phaseoli* exhibit widespread *intI* inactivation, likely occurring early in their divergence, leading to more stable cassette arrays and conserved integron-associated phenotypes. The number and diversity of genes within cassette arrays varied significantly by species and, to a lesser extent, by the ecological context of plant host cultivation. While most cassettes encoded proteins without a known function, those with annotated roles were associated with stress response mechanism, competitive exclusion and plant-associated functions. Together, our findings demonstrate that integrons in *Xanthomonas* likely originated from a single ancient acquisition event, preceding genus-wide speciation, and have co-evolved with *Xanthomonas* pathovars as they adapted to distinct plant hosts.

**Impact Statement**

This study provides the first comprehensive genus-wide analysis of integron evolution and dynamics in *Xanthomonas*, a globally distributed and versatile plant pathogen. Integrons are genetic platforms that allow horizontal transfer of genes. They are well characterized in human pathogens where they mediate transfer of antibiotic resistance genes, but little is known about their role in plant-associated bacteria. We showed that in *Xanthomonas*, the integron platform was ancestrally acquired, yet integrons have undergone repeated lineage-specific inactivation events. Despite widespread erosion of integron activity, some species such as *Xanthomonas campestris* maintain robust and functionally diverse integrons that continue to shape genome plasticity. Notably, high cassette diversity, combined with the presence of rare and often uncharacterized genes within these arrays (some potentially involved in environmental sensing or host interaction), suggests that integrons may serve as reservoirs of adaptive potential. Our findings reshape current views of integron function beyond antibiotic resistance and highlight their long-term role in microbial evolution, niche adaptation and genome innovation in plant-associated bacteria.

## Data Summary

The authors confirm that all supporting data, code and protocols have been provided within the article or through supplementary data files. Accession numbers of the genomes analysed in this manuscript are listed in Table S1 (available in the online Supplementary Material).

## Introduction

Integrons are genetic elements that facilitate horizontal gene transfer (HGT) in Bacteria [1] and Archaea [2]. They are ancient structures that have been involved in the evolution of bacterial genomes for hundreds of millions of years [3]. Functional integrons are composed of an integron-integrase gene (*intI*), an integron recombination site (*attI*) and a promoter that drives the expression of the integrase (P_int_) and generally also have a second promoter (P_c_), oriented for the expression of gene cassette array proteins [4,6].

Gene cassettes are mobile, non-replicating elements, which generally consist of an ORF and an *attC* site. They exist as circular molecules when excised from a cassette array. Although cassettes generally consist of a single ORF, cassettes that have two ORFs or ORF-less cassettes with promoter activity have been observed [7,9]. The tyrosine recombinase IntI can capture circular cassettes and integrate them into the genome by mediating the recombination between *attC* and *attI* sites, forming arrays of 1 to >200 gene cassettes. As well as mediating the insertion of cassettes, IntI can also excise cassettes from an array. Cassette arrays lacking an integrase are called CALIN (clusters of *attC* sites lacking integron-integrase) [10] and can be mobilized in trans by a functional IntI.

Technically, integrons themselves are not mobile, whereas their gene cassette cargo is. Integron activity can facilitate horizontal gene cassette transfer between prokaryotes, contributing to the rapid evolution and genetic diversity of Bacteria and Archaea [4]. Because DNA damage and nutrient starvation induce the activity of the integron integrase via bacterial SOS and stringent responses [11,13], acquisition of novel genetic functions is preferentially triggered during periods of environmental challenge. This provides a mechanism for adaptive innovation when survival pressures are highest.

Integrons have been extensively studied because of their role in the accumulation and dissemination of antibiotic resistance genes in clinical isolates [14,15]. However, integrons are also common in other environments such as marine environments [16], soil [17] and the plant rhizoplane [18,19]. In these environments, the majority of gene cassettes remain functionally uncharacterized, but predicted functions encode extensive functional diversity including traits involved in microbe–host interactions [4,20, 21].

*Xanthomonas* is a genus of Gram-negative bacteria in the class *Gammaproteobacteria* that contains pathogenic strains able to cause disease in more than 400 different plants. In common with other genera of plant pathogens, *Xanthomonas* also contains strains associated with plants that do not cause disease symptoms [22,24]. *Xanthomonas* is a large genus that encompasses more than 35 species, which are subdivided into pathovars [25].

Integrons have been described in some *Xanthomonas* isolates [26,28]. Using PCR with site-specific primers annealing to the proximal part of *intI* and to the *attI* region, Gillings and colleagues screened 32 *Xanthomonas* strains representing 12 pathovars and found that all the strains analysed had an integron. In all cases, *intI* was integrated downstream of the acid dehydratase gene, *ilvD*; however, the majority of integrase genes were predicted to be inactivated by frameshifts, stop codons or large deletions. Groups of strains with the same deletions or stop codons/frameshifts in *intI* usually contained identical arrays of genes. There was no evidence of integrons generating diversity within pathovars but strong evidence for diversity between pathovars. With some minor exceptions, individual pathovars had distinct proximal gene cassette arrays, and every cassette identified was found only in one pathovar.

Complete genome sequences for hundreds of *Xanthomonas* isolates are now available, allowing for a systematic and detailed investigation of integrons in these important plant-associated bacteria. Here, we have examined the presence of integrons in all publicly available complete genomes of *Xanthomonas*, significantly expanding our knowledge of these elements and showing how integrons have been a dynamic component during *Xanthomonas* genome evolution.

## Methods

### *Xanthomonas* classification and phylogeny

Complete genomes of *Xanthomonas* were downloaded from the National Center for Biotechnology Information (NCBI) database (NCBI txid: 338) (708 genomes, last accessed September 2024) (Table S1). Taxonomic classifications of the genomes were based on the Genome Taxonomy Database (GTDB) [29] release 2.2.0 using GTDB-Tk v2.4.0 [30] (Table S1). The command classify_wf was used with default settings. GTDB-Tk first aligns 120 single-copy phylogenetic marker genes and then classifies each genome based on its placement into domain-specific reference trees (built from 47,899 prokaryote genomes), its relative evolutionary divergence and average nt identity (ANI) to reference genomes in the GTDB. Two genomes (GCA_041228075.1 and GCA_002240395.1) did not belong to the *Xanthomonas* genus (*Luteibacter* and *Luteimonas*_D, respectively) and were removed from the dataset. The GTDB-Tk classification system does not strictly adhere to the traditional nomenclature of *Xanthomonas* strains, sometimes reassigning species. We use GTDB-Tk classification in order to use a standardized microbial taxonomy method based on genome phylogeny and not to attempt to re-classify or rename isolates.

Phylogeny of the *Xanthomonas* genus was inferred using the alignment of the 120 marker genes produced with the GTDB-Tk command classify_wf using GTDB-Tk command infer (default setting with --gamma) that uses FastTree 2.1 [31] to build an approximately-maximum-likelihood phylogenetic tree. The tree was rooted with GCA_041228075.1 (*Luteibacter*), and then, the tip was removed with the R package ape 5.8-1 [32]. Single-species phylogeny was built using Realphy [33], which maps genomes to a series of reference genomes via bowtie2 2.0.0-beta7 [34]. From these, multiple sequence alignments are constructed and used to infer phylogenetic trees via PhyML 3.1 [34]. Trees are visualized using the ggtree 3.16 [35] and ggtreeExtra 2.3 [36] R packages.

### Analysis of integrons

Integrons were identified with IntegronFinder 2.0.5 [37] (parameters: --local-max --gbk --promoter-attI) (Table S1). IntegronFinder 2.0.5 was run with default --calin-threshold (default: 2), and putative CALINs were identified if they carried at least two *attC* sites. To determine the genomic location of the integrons, identified integron sequences were extracted together with 5 kb of flanking regions upstream and downstream (fasta files deposited at https://github.com/EC-MQ/Xanthomonas_integrons) and annotated with Bakta 1.11.3 [38]. Taxonomic origins of integron gene cassette recombination sites (*attC*s) were predicted using the script attC-taxa.sh [39,40]. The *attC*-taxa pipeline (https://github.com/timghaly/attC-taxa) can detect *attC* sites that are conserved among 1 of the 11 bacterial taxa, comprising *Alteromonadales*, *Methylococcales*, *Oceanospirillales*, *Pseudomonadales*, *Vibrionales*, *Xanthomonadales*, *Acidobacteria*, *Cyanobacteria*, *Deltaproteobacteria*, *Planctomycetes* and *Spirochaetes*.

*IntI* sequences identified by IntegronFinder were aligned using PRANK [41] run with default settings, the alignment was stripped of gaps with the goalign ‘clean sites’ command [42] and the tree was inferred with FastTree 2.1 [31] using the generalized time-reversible model (--gtr).

We used multiple approaches to annotate the cassette-encoded proteins. Proteins were functionally annotated with eggNOG-mapper v2 [43,44], executed in DIAMOND [45] mode. Additionally, Foldseek [46] was used to create a protein structural database with the ProstT5 protein language model [47]. The database was then used to perform structural alignment against the Swiss-Prot database using a Foldseek search. Foldseek convertalis was used to convert the alignment database in a tab-delimited output, and only functional annotations against the Swiss-Prot database with a coverage of 80% and an e-value <0.001 were considered.

To identify genes involved in functions of ecological relevance (trace gas oxidation, carbon cycling, nitrogen cycling, sulphur cycling, phosphorus cycling, iron cycling, plant–microbe interactions and osmotic stress tolerance), we used the *EcoFoldDB-annotate* pipeline [48].

Plant growth-promoting traits were identified using the Plant Growth-Promoting Traits Prediction (PGPT-Pred) tool [49]. SignalP v6.0 [50] was used for signal peptide predictions as a marker for identifying transmembrane or secreted gene products. DefenseFinder [51] was used to detect known anti-phage systems.

Unique gene orthologues encoded by the cassettes and their distributions were identified with Proteinortho 6.0.22 [52] run with the -singles option.

To detect genes encoding type III secretion effectors (T3SEs), we used a database of 66 previously characterized *Xanthomonas* T3SEs retrieved from the EuroXanth platform [53,54]. T3SEs in the cassettes were identified with Proteinortho v6.0.22 [52] by querying the curated effector database against the amino acid sequences of the cassette-encoded genes.

Phylogenetic signal in the number of *attC* sites across genomes was first assessed with Pagel’s λ tests using the R package caper 1.0.3 [55]. To evaluate the influence of cultivation type on *attC* site number while accounting for shared evolutionary history, phylogenetic generalized least squares regression was conducted using the R package nlme 3.1 [56].

### HGT events between species

Alfy 1.0.5 [57] was used to guide the detection of highly similar cassettes present in the integron arrays. Alfy was run with the -M option, selecting only matches with a *P* value <0.05 within a sliding window of 100 bp. Only arrays sharing the closest homology (recombination), with at least 10% of their array sequence, were considered. The output of this was further filtered, using ANI comparisons to assess the likelihood that shared cassettes represented probable HGT events. FastANI 1.33 [58] was run between the arrays and the whole genomes of the strains. We classified an event as HGT when the ANI between arrays exceeded 96%, the ANI between the genomes harbouring them was below 95% and the array ANI was at least 2% higher than the genome ANI. Cytoscape [59] was then used to visualize the numbers of cassettes transferred between *Xanthomonas* species.

## Results and discussion

### The *Xanthomonas* integron platform is ancestral

*Xanthomonas* complete genomes classified based on the GTDB as ‘*Xanthomonas*’ (*n*=629) and ‘*Xanthomonas_A*’ (*n*=78), known also as *Xanthomonas* groups 2 and 1, respectively [60], were analysed for integron features (Table S1). We retained the genomes of *Xanthomonas_A*, even though they are classified as a different genus by GTDB-Tk, because from a plant pathology perspective, they are still considered *Xanthomonas* and encompass *Xanthomonas translucens*, an important causal agent of diseases in cereal crops and forage grasses [61].

**Fig. 1.**
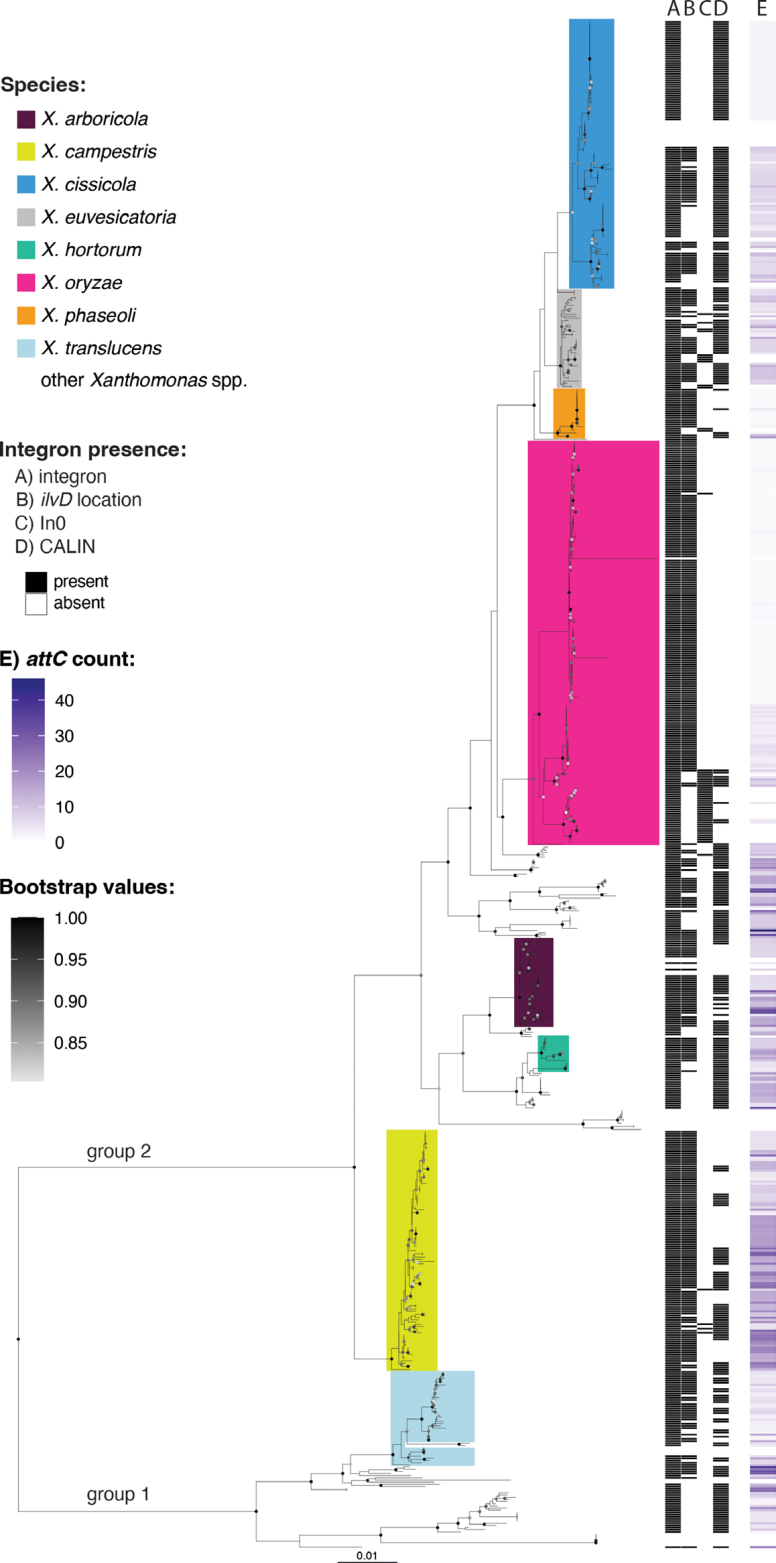
Distribution of integron platforms in *Xanthomonas* groups 1 and 2. The maximum likelihood phylogenetic tree is based on a concatenated alignment of 120 top-ranked marker proteins. The tree was rooted at the midpoint. Tips are labelled with species names assigned with GTDB phylogeny. Coloured clades show species with more than genomes. A) Integrons were classified as present if the isolates carried a functional integron, *intI* only or an *attC* array only. B) indicates the presence of a complete integron or *attC* sites in the *ilvD* locus. C) indicates the presence of an *intI* lacking cassettes (In0). D) indicates the presence of CALINs (cluster of *attC* sites) not in the *ilvD* locus. E) indicates the number of *attC* sites (number of cassettes) in the genome. Only bootstrap values 0.80 are shown. Scale bar indicates substitutions per site.

Using IntegronFinder 2.0.5 [37], we identified integrons, In0 (integron-integrases that lack cassettes) or CALINs in 93% of the genomes (657 genomes) (Fig. 1, Table S1). *Xanthomonas albilineans* (*n*=6, group 1) and *Xanthomonas fragariae* (*n*=6, group 2) were the only species with more than two genome sequences available that did not harbour either an *intI* or *attC* sites. However, integrons have been previously detected in some isolates of *X. fragariae* via PCR amplification [26].

Because integrons are widespread in *Xanthomonas*, our first question was whether the acquisition of the integron module was ancestral. The integron-integrase encoding gene *intI* was integrated downstream of *ilvD* in all genomes, with the two exceptions being isolates of *Xanthomonas oryzae* and *Xanthomonas cucurbitae*. Of the 187 *oryzae* isolates examined, *X. oryzae* pv. *oryzicola* (26 genomes) harbour a truncated *intI* (561 bp) that was not integrated downstream of *ilvD*. However, the integron locus (*intI* and cassette array) is surrounded by transposases, which could indicate that the integron was moved via transposition or could be a result of assembly errors, which are common in regions flanked by insertion sequences. In all seven *X. cucurbitae* genomes, *intI* was not integrated downstream of *ilvD*. However, downstream of *ilvD*, there are two genes without recognizable *attCs*, which are encoded in the other integrons (including a DUF1488 encoding gene which occurs in multiple species).

The different genomic locations of *intI* could be explained by either within-genome transposition or recombination (*intI* was initially located downstream of *ilvD* and then moved), or by an independent acquisition of *intI* from another source, which was integrated in a different locus. To test this, we constructed the phylogeny of all the *Xanthomonas intIs* (Fig. S1, Table S2). If *intIs* located elsewhere from *ilvD* were acquired independently, they would likely have a different evolutionary history and form separate clades in the *intI* phylogeny.

The phylogeny of *intI* clusters this gene within species, generally being independent of the integration locus. *X. oryzae intIs* cluster together, and the *X. cucurbitae intIs* form a clade with pv. *phaseoli* (Fig. S1). The only notable exception is *Xanthomonas arboricola. X. arboricola* strains can carry one of the two variant *intIs*. In two strains, *intI* clusters closest to the *Xanthomonas campestris intIs* are truncated and likely not functional. Phylogenetically close *X. arboricola* strains do not harbour an *intI* at all. The other *X. arboricola* strains carry *intI* (either full-length or with an early stop codon) that are more distantly related to all other *intIs*. These more distantly related *intI* could have been acquired from a source outside *Xanthomonas*. We compared the *ilvD-intI* locus sequence in two representative *X. arboricola* strains harbouring the two *intI* variants (GCA_018141705.1 and GCA_905367745.1). While the *ilvD* genes share 97.1% ANI, the *intIs* share 68.2% ANI. This discrepancy in ANI values between two adjacent genes suggests a recombination event [62], with abrupt shifts in ANI at gene boundaries a common indicator of a recombination breakpoint.

**Fig. 2.**
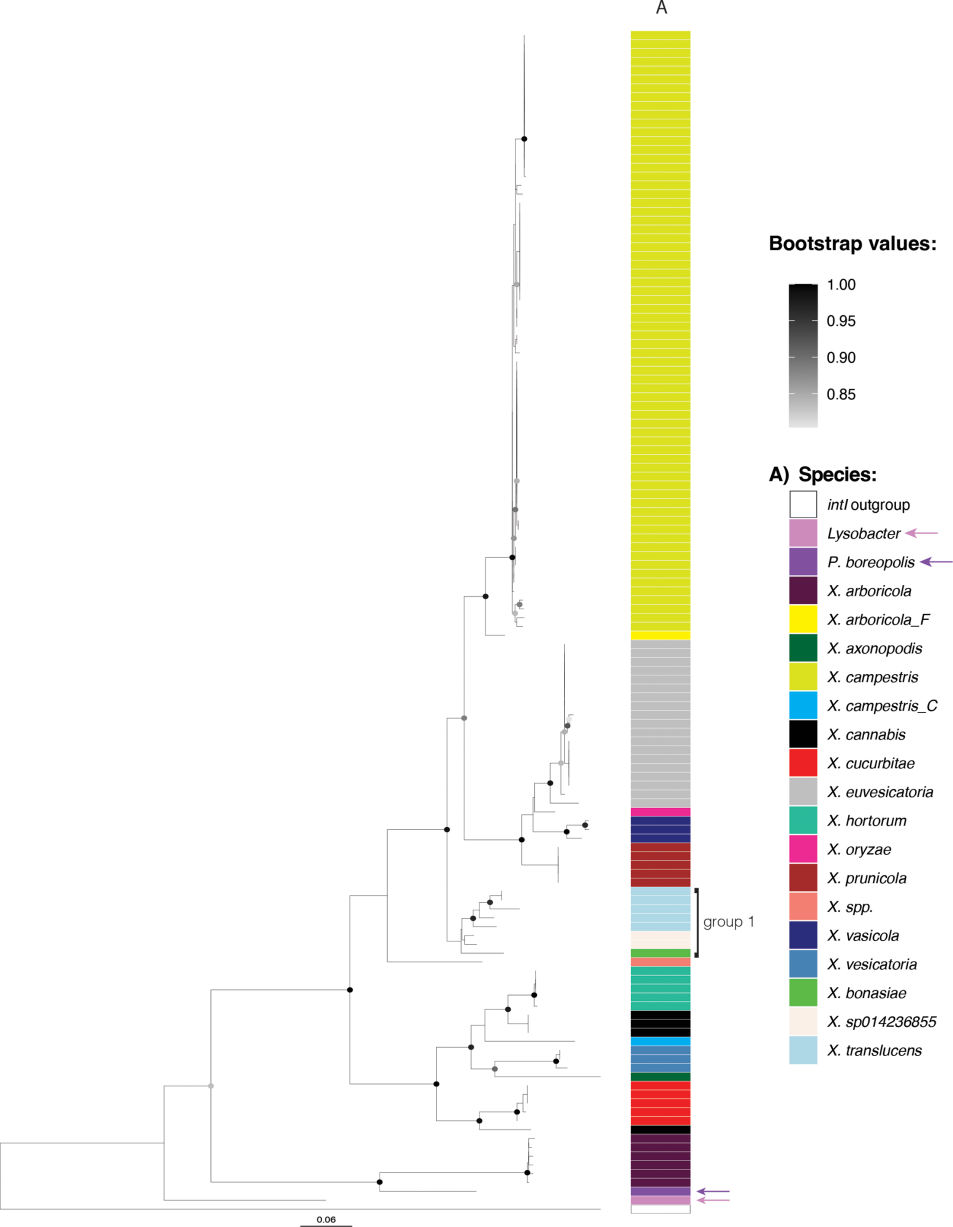
Phylogeny of *intI*. Sequences of *intI* longer than 900 bp were aligned with PRANK, stripped of gaps and used to infer the tree with FastTree. *intI* from *Vibrio* sp. SCSIO 43136 (locus tag: J4N39_08275) was used as an outgroup. *intI* from group 1 *Xanthomonas* are indicated on the side of the plot; arrows indicate *intI*s of *Lysobacter* sp. CECT 30171 and [*Pseudomonas*] *boreopolis* strain GO2. The scale bar indicates substitutions per site. Only bootstrap values 0.80 are shown.

We used two full-length *intI* variants (one belonging to *X. arboricola* and one to *X. campestris*) as a query in a blastn search on complete genomes in the NCBI excluding *Xanthomonas* (taxid: 338). The two closest *intI*s belonged to *Lysobacter* sp. CECT 30171 (locus tag: LYB30171_00805, 93% coverage and 74% identity against *X. campestris intI*) and [*Pseudomonas*] *boreopolis* strain GO2 (locus tag: M3M27_18655, 92% coverage and 91% identity against *X. arboricola intI*) (both genera in the *Xanthomonadaceae* family). Curiously, in isolate GO2, *intI* was also located downstream of *ilvD*. We constructed a phylogenetic tree using *Xanthomonas intI* sequences longer than 900 bp and included the *intIs* of CECT 30171 and GO2, using *Vibrio* sp. SCSIO 43136 *intI* as an outgroup (Fig. 2). The *intI* variants in *X. arboricola* were more closely related to the *intI* in GO2 than the other *Xanthomonas intIs. ilvD* phylogeny instead clusters together all *X. arboricola* isolates within the *Xanthomonas* group 2, and GO2 as basal to both *Xanthomonas* group 1 and 2 isolates (Fig. S2). This suggests that, in those *X. arboricola* strains, *intI* was acquired via HGT from another member of the *Xanthomonadaceae* family.

The phylogeny of *ilvD* and *intI* is incongruent, with the *ilvDs* of *Xanthomonas* group 1 forming a separate group in comparison to group 2 (Fig. S2), while the group 1 *intIs* are nested within the most common *Xanthomonas intIs* (Fig. 2). Genetic exchange between a restricted number of strains of group 2 and the entire group 1 clade has been reported [60]; therefore, it is plausible that horizontal transfer of an *intI* gene from a group 2 *Xanthomonas* species occurred during the early evolutionary history of group 1 *Xanthomonas*.

Together, this suggests that the acquisition of the integron platform was ancestral to the *Xanthomonas* genus, and then, *intI* diversified with the species. The exceptions are some *X. arboricola* strains that could have recruited *intI* from another source, while group 1 *Xanthomonas* could have acquired *intI* from group 2 *Xanthomonas* in one single-gene flow event.

Additionally, ~50% of genomes harbour CALINs, here defined as clusters of at least two *attC* sites, in loci other than the canonical *ilvD* region and in the absence of a proximal *intI* gene. In genomes where *intI* is retained, this gene is consistently integrated downstream of *ilvD,* while these CALINs show spatial separation, being located in other regions of the genome. Among the species represented by more than ten genomes, CALINs not adjacent to *ilvD* were identified at seven distinct chromosomal loci. In *X. arboricola*, *X. campestris*, *X. cissicola*, *X. euvesicatoria* and *X. hortorum*, CALINs consistently occurred adjacent to *lamG* and/or *secF*. In *X. cissicola*, additional CALINs were located near *recD* and a gene encoding an NAD(P)/FAD-dependent oxidoreductase. In *X. oryzae*, two strains possessed CALINs adjacent to an outer membrane protein gene, while in *X. translucens*, CALINs were observed at two distinct genomic positions. One *X. campestris* strain (GCA_028749605.1) harboured a CALIN on a conjugative plasmid. The relatively conserved positioning of CALINs across species supports the hypothesis of ancestral integron activity. This contrasts with experimental data from *Escherichia coli*, where cassette libraries integrated via *attG* sites (consensus recombination sequences that are not *attC* or *attI*) exhibited a broad distribution across numerous genomic loci, indicating that *de novo* integration is typically less site-specific [63].

### Acquisition of the integron platform is ancestral but its activity is progressively lost

**Fig. 3.**
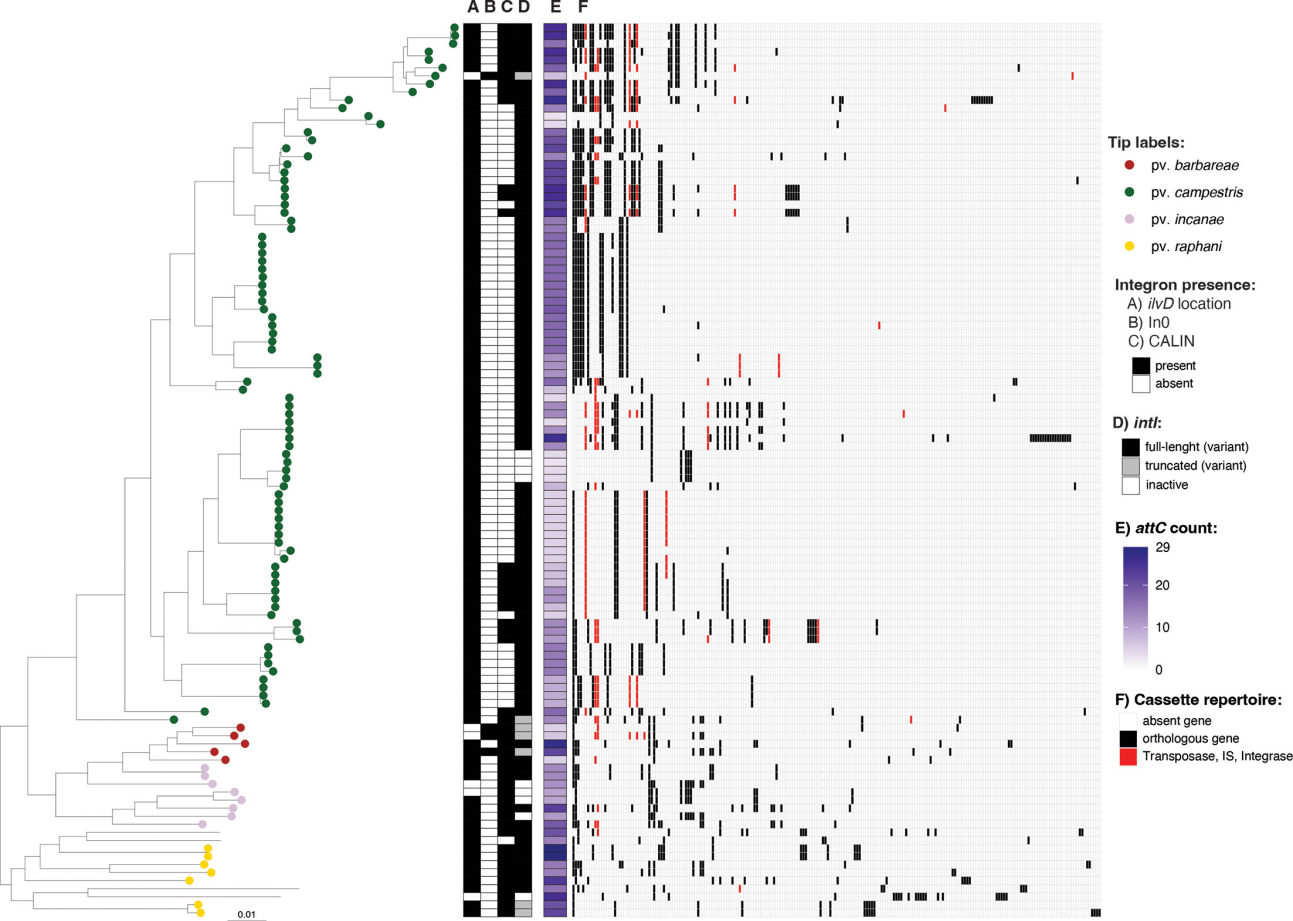
Integrons in *X. campestris*. The phylogeny of *X. campestris* was built using Realphy, using GCA_000007145.1 as a reference genome. The tree was rooted with GCA_000972745 (*X. arboricola*); then, the tip was removed from the tree. Tip labels show pathovars, which were assigned from the literature search (Table S3). The scale bar indicates substitutions per site. A) indicates the presence of a complete integron or *attC* sites in the *ilvD* locus. B) indicates the presence of an *intI* lacking cassettes (In0). C) indicates the presence of CALINs (cluster of *attC* sites) not in the *ilvD* locus. D) indicates whether IntI is predicted to be functional (full length) (Table S3). E) indicates the number of *attC* sites (number of cassettes) in the genome. Panel F) represents the distribution of orthologous genes among all cassettes carried by the corresponding isolate inferred with Proteinortho. Orthologous genes are listed in decreasing order based on the number of strains within the species that carry them.

**Fig. 4.**
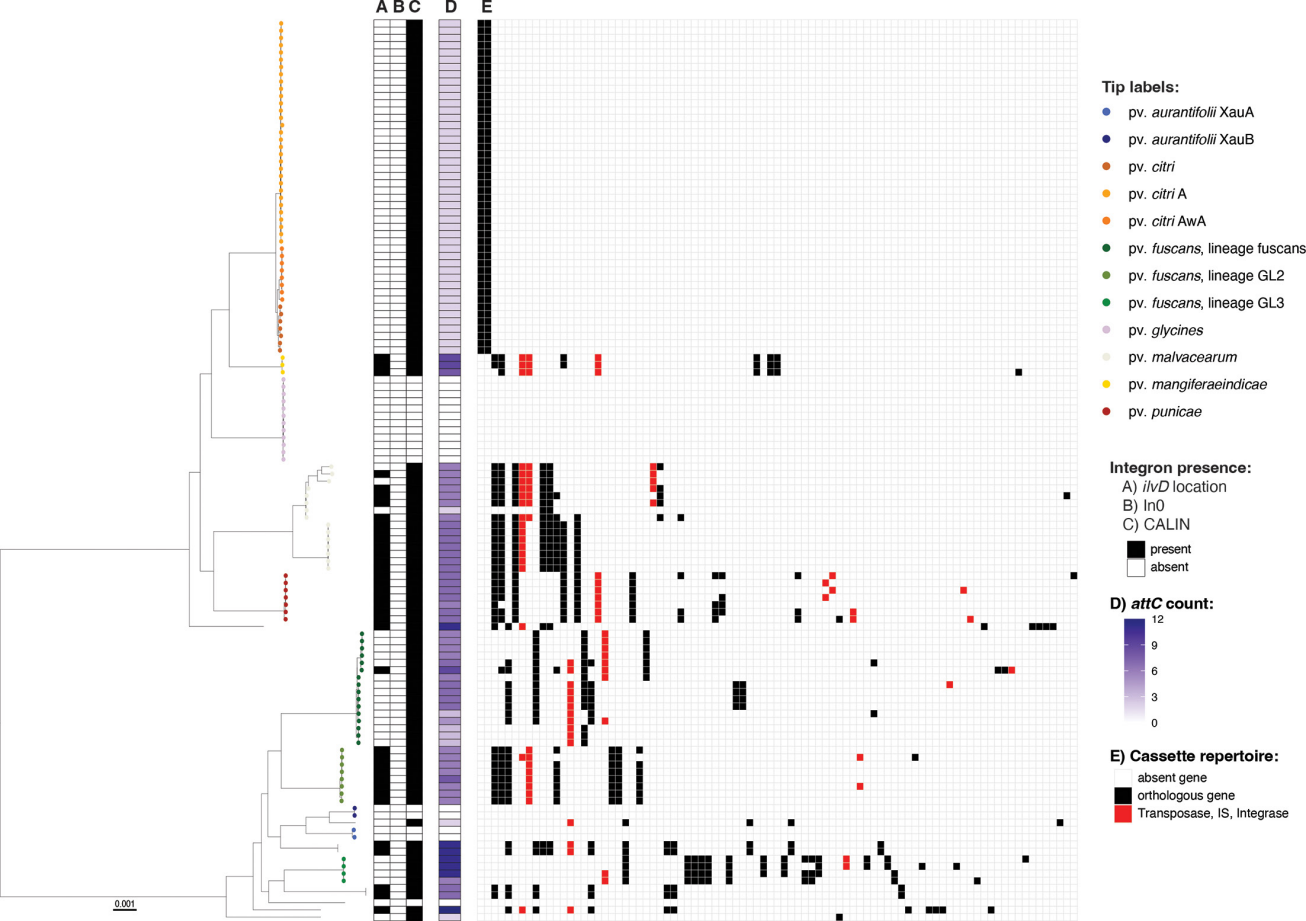
Integrons in *X. cissicola*. The phylogeny of *X. cissicola* was built using Realphy, using GCA_000007165.1 as a reference genome. The tree was rooted with GCA_000009165.1 (*X. euvesicatoria*); then, the tip was removed from the tree. Tip labels show pathovars, which were assigned from the literature search (Table S3). The scale bar indicates substitutions per site. A) Indicates the presence of a complete integron or *attC* sites in the *ilvD* locus. B) indicates the presence of an *intI* lacking cassettes (In0). C) indicates the presence of CALINs (cluster of *attC* sites) not in the *ilvD* locus. D) indicates the number of *attC* sites (number of cassettes) in the genome (Table S3). Panel E) represents the distribution of orthologous genes among all the cassettes carried by the corresponding isolate inferred with Proteinortho. Orthologous genes are given in decreasing order based on the number of strains within the species that carry them.

To investigate integron integrase activity and the complement of integron gene cassettes in *Xanthomonas* genomes, we focused on species which had more than ten genomes in our dataset: *X. campestris* (*n*=111) (Fig. 3), *X. cissicola* (*n*=124) (Fig. 4), * X. arboricola* (*n*=41) (Fig. S3), *X. translucens* (*n*=46) (Fig. S4), *X. euvesicatoria* (*n*=46) (Fig. S5), *X. oryzae* (*n*=161) (Fig. S6), *X. phaseoli* (*n*=23) (Fig. S7) and *X. hortorum* (*n*=12) (Fig. S8). A detailed description of the integrons in these eight species is reported in the Supplementary Results Section.

Although integron-associated sequences are common in *Xanthomonas* groups 1 and 2, most strains appear to have lost integron integrase activity (*intI* is either truncated or absent), and the distribution of functional integrons appears to be species-specific. However, among the species analysed, *X. campestris* stands out for maintaining a largely functional integron platform (Fig. 3). A high proportion (85.6%) of its genomes carries a full-length *intI* gene, and integrons are consistently flanked by extensive cassette arrays (ranging from 3 to 29 *attCs*, averaging 14.3 per genome). Cassette composition is highly variable even among strains of the same pathovar, suggesting that, in *X. campestris*, integron activity drives rapid diversification within genetically related groups. This robust activity is also evident in the gene content: 216 orthologous cassette-encoded genes were identified, 41.1% of which are singletons (observed in only 1 instance in this dataset). Despite this extensive variability, a gene encoding a DUF1488 domain-containing protein is present in the cassettes of 81.5% of genomes, while *symE* (a toxin-encoding gene) is found in 56.8% of the cassette arrays. This level of integron retention suggests that active integrons continue to play a key role in the adaptive evolution of *X. campestris*.

*X. arboricola* exhibited a modest preservation of the *intI* gene with 7% of strains retaining a full-length *intI*. In the remaining genomes, *intI* is either deleted or contains an early stop codon. The *X. arboricola* phylogeny divides strains into two clades, clade A which includes pathovars that cause diseases in *Prunus*, *Juglans* and *Corylus* spp. (pvs. *pruni*, *juglandis* and *corylina*, respectively) and clade B which comprises many isolates lacking metadata (Fig. S3). Among clade A isolates, none possess a full-length *intI*. In contrast, clade B strains that carry *intI*, either full-length or truncated (accounting for 58.8% of clade B isolates), harbour a variant likely acquired via HGT, suggesting the acquisition event dated prior to the diversification of this clade. Strains of clade B also carried more cassettes and a broader array of genes compared to clade A.

*X. translucens* (Fig. S4) and *X. euvesicatoria* (Fig. S5) emphasize the dynamic nature of integrons in *Xanthomonas*. Both species exhibit high frequencies of full-length *intI* (22.7% and 45.6%, respectively), and variability in cassette content is high (44.6% and 50%, respectively).

*X. oryzae* includes pathogens of rice (pv. *oryzae* and pv. *oryzicola*) and pathogens of a pervasive weed species that grows along rivers and canals surrounding rice paddies (pv. *leersiae*). However, only X11-5A, a weakly pathogenic strain [64] distantly related to the above-mentioned pathovars, carries a full-length *intI*. Divergent cassette compositions were observed among the pathovars (Fig. S6). This supports the hypothesis that integron activity in *X. oryzae* was ancestrally active, followed by progressive inactivation concomitant with pathovar diversification.

None of the *X. cissicola* (Fig. 4) and *X. phaseoli* (Fig. S7) isolates retain a full-length, functional *intI*. However, 28.7% and 38% of the genes carried by the cassettes in these species, respectively, appear in one isolate only (singletons). *X. cissicola* comprises isolates commonly known as *X. citri*, an important pathogen able to infect many plants including *Citrus* (pv. *citri*). *X. citri* pv. *citri* includes three recognized pathotypes: A, A* and AwA. All pv. *citri* genomes analysed here, which include both A and AwA pathotypes, harbour a CALIN element adjacent to *secD* and lack the integrase gene *intI*. All the CALINs in the pv. *citri* contain the same two gene cassettes, which are not present in any other genome analysed in this study, suggesting that the integron platform was inactivated prior to pathotype diversification (Fig. 4). A recent genomic study of 95 pv. *citri* strains estimated that the diversification of these pathotypes occurred 1,730 to 5,663 years ago [65]. Given that citrus domestication is thought to have occurred at least 2,000 years ago [66], the inactivation event likely predates the domestication of the host genus. One of the two cassettes encodes a protein homologous to members of the late embryogenesis abundant (LEA) protein family, which is known to confer protection against water deficit in bacteria [67]. This indicates that the cassette’s function could be oriented toward general environmental stress rather than mediating specific interactions with the plant host.

### Gene cassette abundance is species-specific

We investigated whether variation in integron cassette array size, quantified by the number of *attC* sites per genome (Table S1), is primarily shaped by phylogeny or by the ecological context of isolation, categorized by cultivation type (crop, non-crop, crop tree, or ornamental host plants) (Table S4). We used only the species that had more than ten genomes in our dataset; additionally, we excluded genomes lacking information on the host of isolation and one strain isolated from mud. We first assessed whether variation in *attC* site counts exhibited a phylogenetic signal. Pagel’s λ test indicated a very strong and highly significant phylogenetic signal (λ=0.99, *P*<2.22e-16), indicating that *attC* abundance is strongly structured by evolutionary history. Given this strong phylogenetic structure, we applied a phylogenetic generalized least squares regression to evaluate the effect of cultivation type while accounting for shared ancestry. Isolates from ornamental plants had significantly more *attC* sites (*P*<0.001; mean=12.1), whereas those from tree-crop environments had fewer (*P*≈0.017; mean=3.05). No significant differences were observed for crop (mean=6.01) and non-crop (mean=9.77) isolates (Fig. S9). Overall, these results suggest that while integron array size is strongly shaped by phylogenetic inertia, reflecting higher similarity within rather than between species, ecological factors, such as characteristics of the plant host, may also contribute. The prevalence of a functional integron integrase appears to influence cassette abundance: species with predominantly inactive integrases tend to carry fewer *attC* sites, likely due to gradual cassette loss. However, the evolutionary forces determining why some lineages retain active *intI* while others don’t are unclear.

### Cassette arrays in *Xanthomonas* harbour diverse but poorly characterized gene functions

Prediction of the taxonomic origins of integron *attC*s was performed with a classification tool developed for *attC*s, which uses both sequence and structural homology information [39], to identify matches to known *attC* ‘types’. The method classified all *attC* sites in the *Xanthomonas* strains analysed as originating within the *Xanthomonadales*. Therefore, there is no evidence of long-distance acquisition of cassettes, and the cassettes appear to circulate at least within the *Xanthomonadales* family.

Arrays greatly varied in length across genomes, with a maximum of 46 cassettes in a single genome (*X. arboricola_F* GCA_040182365.1) and 33 cassettes in a single array (*X. arboricola* GCA_041475745.1 and GCA_041475785.1) (Table S1). Despite the majority of the isolates carrying inactive integron modules, with *intI* deleted or truncated, the cassette arrays appear to have been extremely dynamic before the inactivation of the integron integrase.

Overall, the 706 *Xanthomonas* genomes carried 1,004 cassette arrays, with a total of 4,773 cassettes. Within these, Proteinortho [52] identified 1,087 different orthologous genes. Of the 1,087 orthologous genes identified by Proteinortho, 476 (43.8%) were present as singletons, unique to one isolate.

Of the 1,087 orthologous genes, only 11.9% could be classified into a known Clusters of Orthologous Groups of proteins (COG) category [68] (Table S5). This underrepresentation of known COGs among cassette proteins is well known and has been reported for integrons from a range of different hosts and environments [19,69,72]. This may be due to sampling bias in databases, with captured cassettes potentially being from uncharacterized environmental organisms [73] or may be a result of cassette genes being subject to high mutation rates after capture.

Of the most represented functions, excluding unknown and recombination and repair, 1.47% of the total cassette-encoded proteins were predicted to play a role in transcription, 0.9% in amino acid transport and metabolism, and 0.55% in defence mechanisms. To gain further insight into possible cassette functions, we performed both sequence-based homology searching against eggNOG 5.0 [44] and protein structural homology searching against the Swiss-Prot database. From this combined approach, putative functions could only be assigned to 6.8% of the proteins. Of these annotated cassette-encoded proteins, 47.7% were classified either as transposase, integrases or insertion sequences (4.8% of the total). The presence of insertion sequences and transposases within gene cassettes [70], and targeting *attC* sites [74,75], has been frequently observed in past integron studies.

Signal peptides were identified in 11.6% of cassette-encoded proteins, using SignalP v6.0 [76]. Again, transmembrane and secreted proteins are commonly encoded by gene cassettes in Bacteria [72,77] and are hypothesized to help facilitate interactions with their broader environment. Additionally, PGPT-Pred [49] predicted 6.7% of cassette-encoded proteins to have plant growth-promoting traits such as biofertilization, plant signal production and stress control. However, the most frequent category, representing 4.9% of total proteins, was associated with competitive exclusion functions. These included antimicrobial resistance and detoxification functions, and toxin–antitoxin systems, which in a plant pathogenic context could contribute to niche adaptation by enhancing survival in a competitive microbial environment. One gene was classified by the EcoFoldDB [48] pipeline as involved in spermidine production, which can play an important role in plant growth and stress [78].

DefenseFinder [51] identified 20 orthologous genes (1.8%) as genes involved in defences against phages. Twelve different defence systems were detected; these included restriction-modification and abortive infection systems, but also the newly described Kiwa [79] and Shedu defence systems [80].

Using a database of previously characterized type three secretion system effectors (T3SEs) retrieved from the EuroXanth platform [53,54], we identified two T3SEs: AvrBs3 [a transcription activator-like effector (TALe)] in African-like pv. *oryzae* [81] and XopAF2 in three *X. vasicola* isolates. T3SEs are proteins secreted via the type III secretion system (T3SS) that suppresses or induces plant defences. TALes are a particular type of T3SE; once inside the host cell, they translocate to the nucleus where their unique domain of tandemly arranged 34-aa repeats mediates binding to specific promoter elements. Several TALes are known to induce host SWEET sucrose uniporter genes, thereby facilitating sucrose efflux from xylem parenchyma into the apoplasm at the infection sites. TALes may enhance disease by targeting susceptibility genes or may trigger a resistance response, and are therefore important for pathogen host range and virulence [81].

Given that the majority of cassette-encoded proteins are of unknown function and often occur as singletons (observed only in one strain), the phenotypic impact of these cassettes remains unclear. It is uncertain whether they confer a selective advantage or are maintained in the arrays through neutral processes. A cassette that is conserved across multiple species is more likely to encode a beneficial function. The most widespread cassette, identified in 13 species, encodes *symE* and the non-coding small RNA *sRNA-Xcc1. sRNA-Xcc1* is known to be activated by regulatory elements of the T3SS [82], suggesting a potential role in plant host colonization. In *E. coli*, *symE* forms part of the SymE-SymR toxin–antitoxin system, where SymE induces nucleoid condensation, disrupts DNA replication and transcription and causes dsDNA breaks [83]. Regulation in *E. coli* occurs via the cis-encoded small RNA *symR* [84], whereas in the integron cassettes, *sRNA-Xcc1* is located upstream of *symE*, suggesting a divergent regulatory mechanism. Notably, unlike most integron-associated genes oriented to be transcribed from the *P_c_* promoter (typically located within *intI*), both *sRNA-Xcc1* and *symE* are oriented in the opposite direction and not under *P_c_* control.

Excluding transposases, the second most recurrent cassette, shared among eight species, encoded a protein of unknown function. The third most recurrent, present in seven species, included two hypothetical proteins and a gene conferring resistance to bleomycin. Bleomycin resistance genes have been previously identified as enriched in gene cassettes relative to total metagenomes in environmental samples, as shown by both cassette-targeted amplicon sequencing and shotgun metagenomics [77]. One of the recurring cassettes is an ‘empty’ cassette, which can occur multiple times in the same array. This cassette could have promoter activity as it was previously suggested in other species [7,9].

### Detection of inter-species horizontal transfer of cassette arrays

To assess interspecies cassette gene sharing, we examined pairs of species that most frequently share orthologous genes. As expected, species harbouring a greater number of cassettes tend to share more orthologous genes with others. For example, * X. arboricola* and *X. campestris* shared the highest number of orthologous genes (21), with *X. campestris* sharing the most overall (57 orthologous genes across multiple species).

**Fig. 5.**
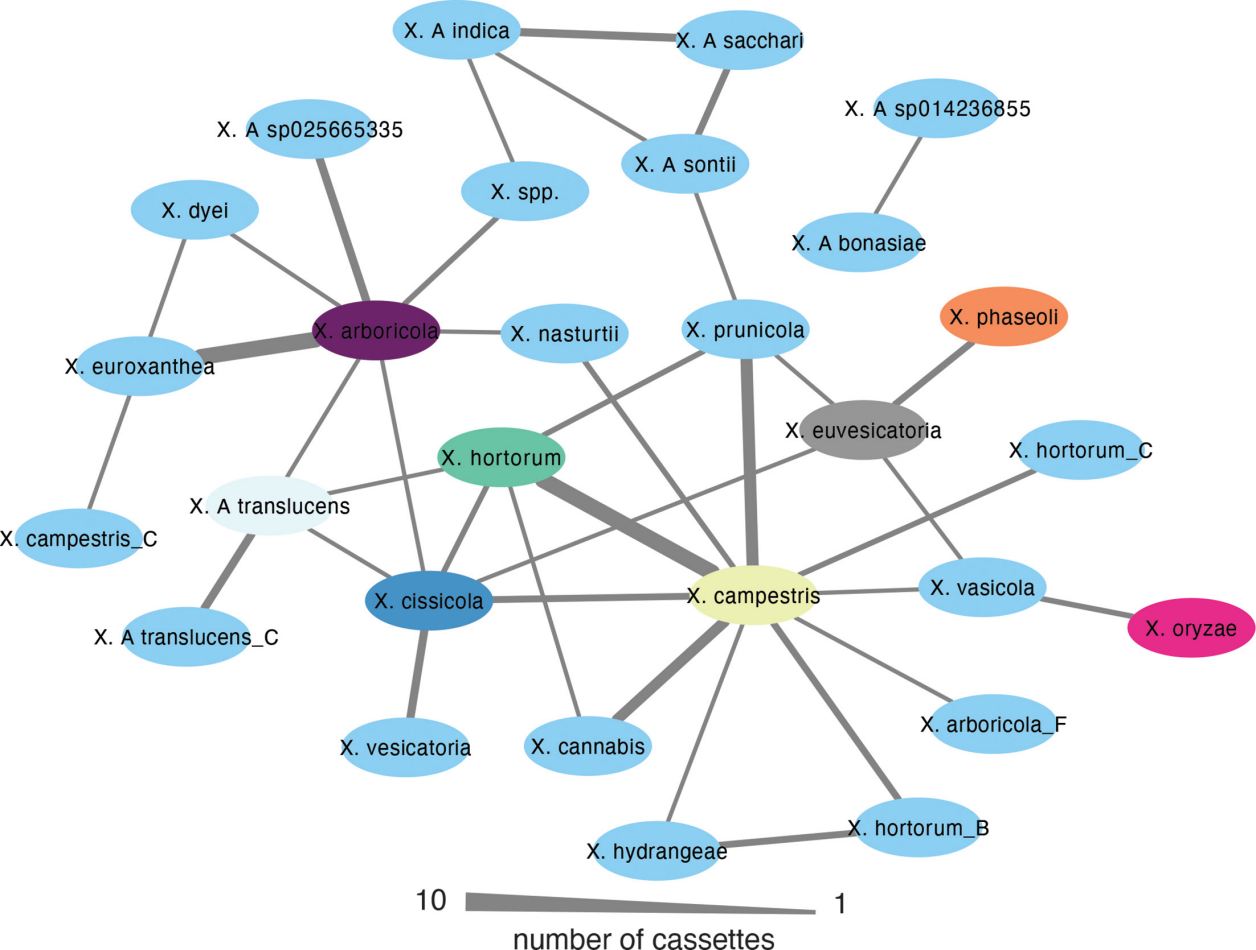
Horizontal transfer of cassettes among *Xanthomonas* species. Cytoscape network illustrating the transfer of cassettes between species. Nodes represent *Xanthomonas* species; species containing more than ten genomes in the dataset are coloured as in Fig. 1 (tip labels). Line width between species is proportional to the number of cassettes moved between species.

We then looked for more recent evidence of HGT of cassettes present in multiple species. We used Alfy v1.0.5 [57] to identify putative regions subject to HGT. We further selected the regions identified by Alfy 1.0.5, selecting those based on the difference in ANI between the cassette and the genomes harbouring them (Fig. S10). With this approach, we detected the movement of 95 cassettes between 38 pairs of species, even between group 1 and 2 *Xanthomonas* (Fig. 5). Fig. 5 shows the number of cassettes exchanged between species, without normalization for genome or cassette abundance. While this approach does not account for differences in genome diversity within and between species, or for uneven sampling depth (i.e. pandemic lineages are often oversampled and appear less diverse in comparison to commensal isolates), it nevertheless provides a direct view of observed horizontal transfer events. The highest flux of cassettes (10) was between *X. arboricola* and *X. euroxanthea* and between *X. campestris* and *X. hortorum*. However, *X. campestris* is the species with the highest flux of cassettes compared to any other species (36). One of the cassettes that showed evidence of horizontal transfer was that carrying *symE* and *sRNA-Xcc1*.

## Conclusions

Our comprehensive analysis of 706 complete genomes reveals that the acquisition of the integron platform is likely an ancestral event in the evolution of the *Xanthomonas* genus. The widespread conservation of the *intI* gene downstream of *ilvD* across species, along with *intI* phylogeny, strongly supports the early acquisition of the integron platform. Only in the *X. arboricola* clade B, the ‘original’ *intI* appears to have been substituted via HGT with an *intI* from another member of the *Xanthomonadaceae* family.

Despite the ancestral acquisition, our results show that integron activity, defined by the presence of full-length, potentially functional *intI* genes, and large and diverse cassette arrays, has been progressively lost in many *Xanthomonas* lineages. This inactivation appears to be species-specific and often predates major evolutionary or ecological transitions, such as pathovar diversification, as seen in *X. cissicola* and *X. oryzae*. Nevertheless, in species such as *X. campestris*, integrons remain robustly functional and continue to contribute to genomic diversification, as evidenced by the presence of full-length *intIs*, high cassette variability and the presence of strain-specific gene cassettes in cassette arrays. Selective pressures and ecological niches may favour the retention of an active integron system in this species and not in the others, for reasons not clear at this time. Loss of integron integrase activity in plant-associated pathovars may be favoured when it stabilizes cassette-associated genetic traits that confer a fitness advantage. Once a beneficial cassette configuration is established, inactivation of the integrase can prevent rearrangements or excisions that would disrupt key functions, enabling these lineages to undergo clonal expansion. Integron integrase activity may also be lost in the absence of positive selection to maintain it. Integrons provide the greatest adaptive advantage in dynamic environments with a diverse pool of accessible gene cassettes. Such diversity is potentially diminished in intensive agricultural systems, where pathogens encounter repetitive host-pathogen cycles and microbial communities are less diverse than in wild plant environments [85]. Under such conditions, the selective pressure to maintain integrase activity might be reduced. In agricultural settings, other types of mobile genetic elements carrying genes already optimized by selection in donor strains may play a more prominent role in driving plant–bacteria evolution [86,90].

The phenotypic impact of the large pool of gene cassettes residing in *Xanthomonas* genomes remains largely unresolved. Nearly half of orthologous genes carried by gene cassettes occurred only once, contributing to strain-level genetic variability. These rare genes may represent a reservoir of adaptive potential but could also be selectively neutral or even transient. Cassettes in *Vibrio* chromosomal integron platforms have been suggested to have been selected to be as neutral as possible [7]. The frequent occurrence of hypothetical proteins, including highly conserved cassette ORFs, suggests important functional roles that remain undiscovered. For instance, a cassette carrying the DUF1488 encoding gene is present in 16.7% of the genomes across six species, sometimes with multiple copies within the same integron array. Among cassette-encoded genes with predicted putative functions, many have predicted functions linked to environmental interaction and thus have the potential to contribute to niche adaptation. For example, the most widespread cassette in our dataset encodes *symE* and the small coding *sRNA-Xcc1*, known to be activated by T3SS regulatory proteins [82], which may influence bacterial growth during plant colonization. In *Xanthomonas*, we found a fairly small fraction of cassette genes to be classified as involved in defence mechanisms, despite the emerging role of integron platforms in encoding anti-phage genes [91,93]. However, this may be an underestimate, as work on large sedentary chromosomal integrons in *Vibrio cholerae* showed that 20% of their cassettes with no predicted function encode for anti-phage defence systems, despite these genes not being detected as such by bioinformatic tools [91].

Importantly, our analysis revealed evidence of interspecies horizontal transfer of cassettes. For both *X. campestris* and * X. arboricola*, there is evidence of recent horizontal transfer of integron-associated cassettes with other *Xanthomonas* species. Interestingly, however, despite their high levels of cassette acquisition, there is little evidence of recent and direct cassette exchange between these two species. Instead, *X. campestris* shows the highest degree of gene sharing with *X. hortorum*, a species more closely related to *X. arboricola* than to *X. campestris*. Conversely, the species with which *X. arboricola* shares the greatest number of cassettes is *X. euroxanthea*, its closest phylogenetic neighbour among the genomes analysed. These patterns suggest that both phylogenetic proximity and ecological overlap, such as co-occurrence in the phyllosphere or endosphere during mixed infections, have contributed to the dynamics of cassette exchange. The shared habitat provides repeated opportunities for interspecies contact and genetic exchange, likely promoting the horizontal dissemination of integron cassettes across the genus.

In summary, our analysis indicates that integron platforms were likely acquired early in the evolution of the *Xanthomonas* genus and have since followed divergent evolutionary trajectories. While many lineages show signs of integron inactivation, others, such as *X. campestris*, retain active systems contributing to genomic diversification. The gene cassettes carried by these integrons, including many with unknown or potentially adaptive functions, add to strain-level variability and may influence niche adaptation. Patterns of HGT suggest that both phylogenetic relatedness and ecological overlap shape cassette exchange across species, highlighting integrons as dynamic elements in the evolutionary landscape of *Xanthomonas*.

## Supplementary material

10.1099/mgen.0.001503 Uncited Supplementary Material 1.

10.1099/mgen.0.001503 Uncited Supplementary Material 2.

## Abbreviations

ANI: average nt identity
CALIN: clusters of attC sites lacking integron-integrase
COG: Clusters of Orthologous Groups of proteins
GTDB: Genome Taxonomy Database
HGT: horizontal gene transfer
NCBI: National Center for Biotechnology Information
PGPT-Pred: Plant Growth-Promoting Traits Prediction
TALe: transcription activator-like effector
T3SE: type III secretion effector
T3SS: type III secretion system
